# iLBE for Computational Identification of Linear B-cell Epitopes by Integrating Sequence and Evolutionary Features

**DOI:** 10.1016/j.gpb.2019.04.004

**Published:** 2020-10-22

**Authors:** Md. Mehedi Hasan, Mst. Shamima Khatun, Hiroyuki Kurata

**Affiliations:** 1 Department of Bioscience and Bioinformatics, Kyushu Institute of Technology, Iizuka, Fukuoka 820-8502, Japan; 2 Biomedical Informatics R&D Center, Kyushu Institute of Technology, Iizuka, Fukuoka 820-8502, Japan

**Keywords:** Linear B-cell epitope, BLAST, Feature encoding, Feature selection, Random forest

## Abstract

**Linear B-cell epitopes** are critically important for immunological applications, such as vaccine design, immunodiagnostic test, and antibody production, as well as disease diagnosis and therapy. The accurate identification of linear B-cell epitopes remains challenging despite several decades of research. In this work, we have developed a novel predictor, Identification of Linear B-cell Epitope (iLBE), by integrating evolutionary and sequence-based features. The successive feature vectors were optimized by a Wilcoxon-rank sum test. Then the **random forest** (RF) algorithm using the optimal consecutive feature vectors was applied to predict linear B-cell epitopes. We combined the RF scores by the logistic regression to enhance the prediction accuracy. iLBE yielded an area under curve score of 0.809 on the training dataset and outperformed other prediction models on a comprehensive independent dataset. iLBE is a powerful computational tool to identify the linear B-cell epitopes and would help to develop penetrating diagnostic tests. A web application with curated datasets for iLBE is freely accessible at http://kurata14.bio.kyutech.ac.jp/iLBE/.

## Introduction

B-cell epitopes (BCEs) are specific regions of immunoglobulin molecules that can stimulate the immune system, which contributes to diagnostic test, antibody production, and vaccine design [1], [2], [3], [4], [5], [6]. B cells are activated by BCEs to perform a variety of biological functions [6], [7], [8], [9], [10], [11], [12]. Identification of BCEs is challenging but crucial for immunotherapy and immunodiagnostics [13], [14], [15], [16]. Nowadays, biopharmaceutical research and development of epitope-based antibodies are growing up due to their high efficiency, biosafety, and acceptability [17], [18]. Thus, the analysis of BCEs is prerequisite for the development of penetrating diagnostic tests and design of the operative vaccines.

BCEs are categorized into two groups: continuous and discontinuous ones [3], [19], [20]. Epitopes in the continuous group, called linear BCEs, consists of consecutive amino acids. Discontinuous epitopes are provided in the form of spatially folded polypeptides and their antigen-binding residues are scattered in their amino acid sequences, making it hard to find them from the primary sequences [21]. To identify the discontinuous epitopes, it is necessary to consider many factors such as biochemical properties and structural proximity [21], [22], [23]. Despite the complex form of the discontinuous epitopes, they are less effective diagnostic/treatment tools than continuous ones [17]. Linear BCEs have vast applications in the area of vaccine design, immunodiagnostic test, and antibody production, as well as disease diagnosis and therapy [24], [25], [26], [27]. Given that experimental identification of BCEs is labor intensive and costly, computational identification of BCEs has gained remarkable interest recently [8], [28], [29], [30], [31]. Several computational approaches have been developed to predict BCEs, which can be categorized into local and global predictors. Local predictors, such as BepiPred [8], Bcepred [32], and COBEpro [26], explore some potential BCE encoding sequences from given protein sequences. These local methods aim to identify the regions or stretchs of proteins that form BCEs [31], but it is difficult to specify the exact regions. Global predictors, such as iBCE-EL [28], IgPred [30], ABCpred [31], SVMTriP [33], and LBtope [34], determine whether a given sequence is a BCE or not. Since the number of BCEs has rapidly increased in the immune epitope database [35], global methods gain attention as the classifier of BCEs. Two global methods, LBtope and iBCE-EL, have recently been developed and publicly available [28], [34]. These two predictors exclusively investigated primary sequence-based features, such as amino acid composition, binary properties, and physicochemical properties, but did not consider any evolutionary information. Therefore advanced analytic tools for identifying linear BCEs are still desirable.

**Figure 1:**
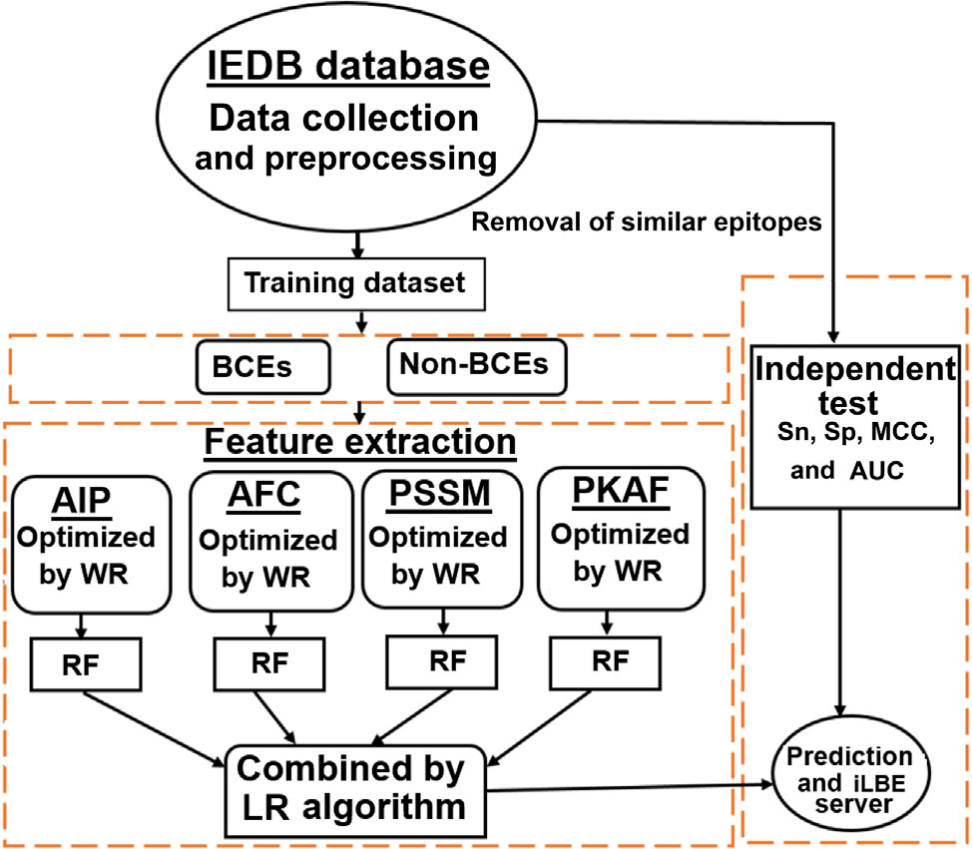
**Overview of iLBE** Firstly, BCE and non-BCE samples were collected from the IEDB database and separated as training and independent datasets. Secondly, the datasets were encoded using four consecutive methods of AIP, AFC, PSSM, and PKAF. Meanwhile, the features were optimized using a non-parametric WR scheme via the RF classifier. After optimization of all parameters, the RF scores for the four features were combined by LR to construct iLBE. iLBE was tested by the independent dataset. iLBE, Identification of Linear B-cell Epitope; BCE, B-cell epitope; IEDB, Immune Epitope Database; AIP, amino-acid index property; AFC, acid frequency composition; PSSM, position-specific scoring matrix; PKAF, profile-based amino acids frequency; WR, Wilcoxon-rank sum; RF, random forest; LR, logistic regression; Sp, specificity; Sn, sensitivity; Ac, accuracy; MCC, Matthews correlation coefficient; AUC, area under curve.

In this work, we have established a computational, global predictor named Identification of Linear B-cell Epitope (iLBE) by integrating sequence and evolutionary features. For evolutionary features, we considered the position-specific scoring matrix (PSSM) and composition of profile-based amino acids frequency (PKAF) encoding descriptors. For primary sequence features, we considered amino-acid index property (AIP) and amino acid frequency composition (AFC). To optimize the consecutive feature vectors, a non-parametric Wilcoxon-rank sum (WR) test was employed. Then the random forest (RF) algorithm using the optimal consecutive feature vectors was used to identify linear BCEs. By the combination of the RF scores through logistic regression (LR), the iLBE yielded better performance than other predictors. Finally we implemented iLBE as a user-friendly web application. The computational outline of the iLBE is shown in Figure 1.

## Method

### Dataset preparation

Experimentally well-characterized datasets of BCEs are needed to develop an accurate machine learning (ML) classifier. We pulled an experimental dataset of linear peptides from the Immune Epitope Database (IEDB), which consists of the verified positive samples (BCEs) and negative samples (non-BCEs) [36], [37]. The IEDB integrates multi-species datasets derived from viruses, bacteria, and fungi. We removed homolog sequences from these collected datasets. To evaluate the potential over-fitting problem in the prediction model, a 70% sequence homology reduction method of CD-HIT was performed [38]. To make a fair comparison with other methods available, the same training and independent samples were retrieved from a recent study [28]. The training model contained 4440 BCEs and 5485 non-BCEs, whereas the independent dataset consisted of 1110 BCEs and 1408 non-BCEs. To avoid the prediction biases, a none-redundant dataset of experimentally validated BCEs and non-BCEs was used, and the samples with more than 70% sequence similarity were excluded. In this study, the peptide length of BCEs and non-BCEs was set to 24. When the length of positive and negative peptide samples was < 24, the null residues (gaps) were added downstream. The curated datasets are shown in our web server and a statistics of the curated datasets is included in Table 1.

**Table 1:** **Statistics of the datasets used in this study**

*Note*: Number and percentage (in the parenthesis) of BCEs and non-NCEs at different lengths in the two datasets are provided. BCE, B-cell epitope; aa, amino acid.

### Feature encoding strategies

#### PSSM profile

The PSSM profile was generated using the PSI-BLAST (a version of 2.2.26+) with the whole Swiss-Prot non-redundant-protein database (a version of December 2010). We used two onset parameters: an iteration times of 3 and E-value cutoff of 0.0001 [39], [40]. The feature vectors were extracted based on the sequence of BCEs and non-BCEs. For each epitope sequence with length 24, a (24 × 20) dimensional vector was generated via the PSSM encoding. When the query peptide length is < 24, zero was added downstream of each PSSM to neutralize the null residues.

#### PKAF encoding

After generating the PSSM profile, we generated PKAF feature vectors [41], [42]. In brief, if the residue pair appears between *m* and *m + k +*1, the composition scores were measured or standardized by the following formula:

(1) $$S_{\mathrm{ij}}=\frac{\sum_{i,j=1}^{T}max\left[\min\left\{PSSM\left(m,x_{i}\right),PSSM\left(m+k+1,x_{j}\right)\right\},0\right]}{W-1}$$

where *W* is the peptide length of BCEs, a *k*-spaced residues characterized as *x_i_{k}x_j_* (*i, j* = 1, 2, …, 20) represent 20 types of common residues, and *T* means that *x_i_*{*k*}*x_j_* performs *T* times for the positive /negative samples. PSSM (*m*, *x_i_*) signifies the score of amino acid *x_i_* at *m*^th^ row in *x_i_*{*k*}*x_j_*, and PSSM (*m* + *k* + 1, *x_j_*) indicates the score of residue *x_j_* at the row of (*m* + *k* + 1)^th^. An optimum value of *k* is 0 or 1, and the dimension of PKAF is 800.

In addition, we employed a similarity-search-based tool of BLAST (version of ncbi-blast-2.2.25+) to examine whether a query peptide belongs to BCEs or not [43], [44]. An E-value of 0.01 via BLASTP was used for the whole Swiss-Prot non-redundant90 database (version of December 2010).

#### AIP encoding

The AIP database (a version of 9.1) contained numerical indices of biochemical and physicochemical properties of amino acids [45]. With assessing various types of indices, we measured 8 types of high informative indices, including NAKH920108, CEDJ970104, LIFS790101, BLAM930101, MAXF760101, TSAJ990101, NOZY710101, and KLEP840101. To produce the feature vectors, the selected AIPs were transformed into the BCEs and non-BCEs. A null residue was used to fill the gap and pseudo residues. In a peptide sequence with length *W*, a (*W* × 8) dimensional vector was generated via the AIP encoding.

#### AFC encoding

The AFC encoding is widely used for representing short sequence peptide motifs [21], [24]. The procedure of AFC is briefly described as follows. When a peptide is composed of 20 types of common residues, it contains (AA, AC, AD, …, YY)_400_ types of residue pairs. An optimal value of *k*, which signifies the frequency of any two-amino acid pairs, was set to 0 or 1. Consequently, 20 × (*k* + 1) × 20 = 800 distinguished residue pairs were generated. The feature vector was then calculated and standardized by the following formula:

(2) $${\left(\frac{N_{\mathrm{AA}}}{N_{\mathrm{total}}},\frac{N_{\mathrm{AC}}}{N_{\mathrm{total}}},...,\frac{N_{\mathrm{YY}}}{N_{\mathrm{total}}}\right)}_{400}$$

where *N*_*total*_ is the length of epitope in the total composition residues. If epitope length *W* is 24 and *k* is 0 or 1, then *N_t_*_*otal*_ = *W* − *k* − 1 is 23 or 22, respectively. (*N_AA_, N_AC_, …, N_YY_*) represents the frequency vector of amino acid pairs within the BCEs and non-BCEs.

### Feature selection

Uncorrelated and redundant features may exist in the generated feature vectors, which can affect the accuracy of a prediction model [40]. Hence, feature selection approaches are important to collect the informative features and to characterize the intrinsic properties of BCEs. To characterize the features important for predicting BCEs, a well-established reduction method of feature dimensionality, WR, was used. A large value of the WR specifies that the corresponding residues have a great impact on the prediction performance. Details in the WR scheme are described elsewhere [39].

### Model training and evaluation

To construct a prediction model, an RF classifier was used. It is a supervised ML algorithm and widely used in bioinformatics research [46], [47], [48], [49], [50], [51], [52]. In brief, the RF is an ensemble of a number of decision trees, H = {H_1_(S), H_2_(S), …, H_N_(S)}, which are built on *N* random subcategories of the training samples. This forest was trained with the bagging method to build an ensemble of decision trees. The general idea of the bagging method is that learning models are assembled to increase the global performance. Details in the RF algorithm were provided in previous studies [39], [48]. The R package was employed to implement the RF into the proposed iLBE (https://cran.r-project.org/web/packages/randomForest/).

Three commonly used ML algorithms, naive Bayes (NB) [53], support vector machine (SVM) [54], and artificial neural network (ANN) [55], were compared with the RF algorithm. The WEKA software [56] was used for the NB and ANN algorithms and the LIBSVM software (https://www.csie.ntu.edu.tw/~cjlin/libsvm/) was used for the SVM algorithm

To construct the final model of iLBE, the respective RF scores evaluated from the four features (PSSM, PKAF, AIP, and AFC) were combined using a LR algorithm. The LR algorithm was effectively used in ubiquitination site prediction [57]. After examining the performance of the resulting S-prediction models (S is the number of the encoding schemes, S = in this study), the final prediction score P was calculated by:

(3) $$log\left(\frac{P}{1-P}\right)=\sum_{n=1}^{S}\beta_{n}R_{n}+\alpha$$

where *β_n_* is the regression coefficient, *R_n_* is the RF score of each feature, and α is the regression constant. The R software package (https://cran.r-project.org/) was employed for a generalized model of LR.

### Performance assessment

To examine the performance of iLBE, four widely-used statistical measures, represented as sensitivity (Sn), specificity (Sp), accuracy (Ac), and Matthews correlation coefficient (MCC), were defined as:

(4) $$\;\text{Sn}=\frac{\text{n(TP)}}{\text{n(TP)}+\text{n(FN)}}$$

(5) $$\text{Sp}=\frac{\text{n(TN)}}{\text{n(TN)}+\text{n(FP)}}$$

(6) $$\;\text{Ac}=\frac{\text{n}(\text{TP})+\text{n}(\text{TN})}{\text{n}(\text{TP})+\text{n}(\text{FN})+\text{n}(\text{FP})+\text{n}(\text{TN})}$$

(7) $$\;\;\;\;\;\;\;\text{MCC}=\frac{\text{n(TP)}\times \text{n(TN)}-\text{n(FP)}\times \text{n(FN)}}{\sqrt{\text{[n(TN)}+\text{n(FN)][n}\left(\text{TP)}+\text{n(FP}\right)\text{][n}\left(\text{TN)}+\text{n(FP}\right)\text{][n(TP)}+\text{n(FN)]}}}$$

where n(TP), n(TN), n(FP), and n(FN) demonstrate the number of anticipated positive, anticipated negative, unexpected positive, and unexpected negative samples, respectively. Furthermore, we depicted the receiver operating characteristic (ROC) curve (Sn *vs.* 1 − Sp) and measured the area under curve (AUC) values [58], [59].

The prediction performance was assessed using 10-fold cross-validation (CV) test on the training model until no further improvement occurred after each round of optimization parameters. The training dataset was separated into 10 groups, where 9 of the groups were used for training and the remaining one for test. This selection process was repeated 10 times to assess the average performance of the 10 models.

### Model development

To develop the prediction model, we first compiled the training and independent datasets in the same manner as described by Manavalan et al. [28] (see Dataset preparation section). The prediction result was evaluated based on the criterion of whether the indication measure (Sp, Sn, MCC, Ac, or AUC) exceeds a threshold value. The AUC value of the ROC curve was evaluated, with the threshold value of the RF score changed to classify a BCE or non-BCE. The threshold value determines the desirable balance to successfully detect positive and negative BCEs. The true positive rate (Sn) and the false positive rate (1 − Sp) were calculated for each threshold value of the RF scores. The high-, moderate-, and low-level thresholds were determined based on RF scores of 0.485, 0.410, and 0.360, respectively, which corresponded to Sp levels of 0.866, 0.747, and 0.636 in the training set results, respectively.

### Web application and implementation

To provide a prediction service of potential BCEs to the scientific community, an accessible web page of the iLBE was established at http://kurata14.bio.kyutech.ac.jp/iLBE/. The web application was written in various programming languages including Perl, R, CGI scripts, HTML, and PHP. The server takes antigen epitopes written with 20 types of common amino acids in the FASTA format. When the submission job is completed, the server returns the prediction results with a combined RF score of the predicted BCEs in a tabular form to the output webpage with the job ID and a query peptide. Users can save the ID for a future query and the iLBE server stores this ID for a month.

## Results and discussion

### Analysis of positional amino acids

**Figure 2:**
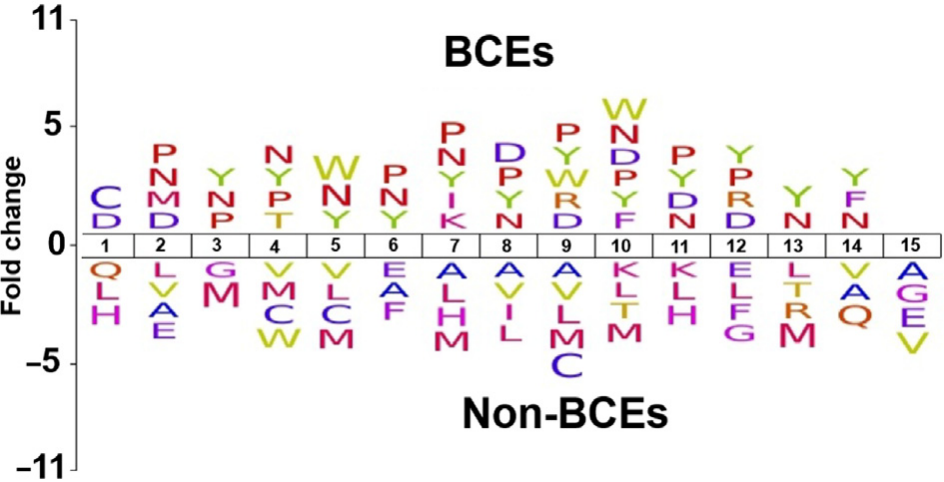
**Distribution of amino acids of BCEs** The iceLogo software (https://iomics.ugent.be/icelogoserver/) is used to present amino acids with a significantly different distribution between BCE and non-BCEs (*P* < 0.05).

To investigate the sequence preference of BCEs and non-BCEs, we performed amino acid positional analysis using the iceLogo software [60]. In the training datasets, 1–15 residues were employed to create iceLogos. The average length of the BCE and non-BCEs was set to 15. Significant differences in the surrounding BCEs and non-BCEs were observed by Welch's *t*-test with *P* < 0.05 (Figure 2). The neutral amino acids P, N, and Y showed a strong preference on BCEs at positions 3, 4, 6, 7, 8, 10, and 11, while amino acids A, H, L, M, and V showed a strong preference for non-BCEs. This analysis supports the idea that different residues are targeted by distinct BCEs, suggesting that combination of different features is critical for accurate prediction of BCEs.

### Selection of the optimal model

To inspect the performance of iLBE, the curated BCE datasets were first coded as mathematical feature vectors based on the four successive encodings of AIP, AFC, PSSM, and PKAF. Given that prediction performance may be impaired by uncorrelated and redundant evidence in the curated features, we used the WR method to optimize the feature vectors. After several trials, top 170, 510, 320, and 490 feature vectors were selected from the AIP, AFC, PSSM, and PKAF descriptors, respectively. Then the selected feature vectors were rearranged in the ascending order of WR values. The RF classifiers were trained by using the final four encoding feature vectors. The decision trees of RF were optimized over the training dataset by a 10-fold CV test. Then the RF scores by the PSSM, AIP, PKAF, and AFC encoding methods were combined by the LR scheme with regression coefficients of 0.435, 0.102, 1.337, and 0.465, respectively. As shown in Table 2, AFC presented a higher performance than any other single encoding approach in terms of Sn, MCC, and AUC in the training dataset. The combined model of iLBE outperformed all the four single encoding approaches in terms of Sn, MCC, Ac, and AUC. The superiority of iLBE was confirmed to be significant by two-tailed *t*-test.

**Table 2:** **Performance comparison among four single feature methods and the combined iLBE**

**Method**	**Sp**	**Sn**	**Ac**	**MCC**	**AUC**	***P*****value**
PSSM	0.703	0.714	0.708	0.368	0.746	0.006
AIP	0.704	0.689	0.697	0.369	0.742	0.006
PKAF	0.705	0.737	0.719	0.429	0.774	0.033
AFC	0.703	0.739	0.719	0.432	0.775	0.038
iLBE	0.747	0.759	0.752	0.496	0.809	

*Note*: A10-fold CV test was applied to the training dataset. A two-tailed *t*-test was performed based on the AUC values, where *P* < 0.05 indicates a significant difference between iLBE and the respective single feature method. PSSM, position-specific scoring matrix; AIP, amino-acid index property; PKAF, profile-based amino acids frequency; AFC, acid frequency composition; Sp, specificity; Sn, sensitivity; Ac, accuracy; MCC, Matthews correlation coefficient; AUC, area under curve; CV, cross-validation.

**Figure 3:**
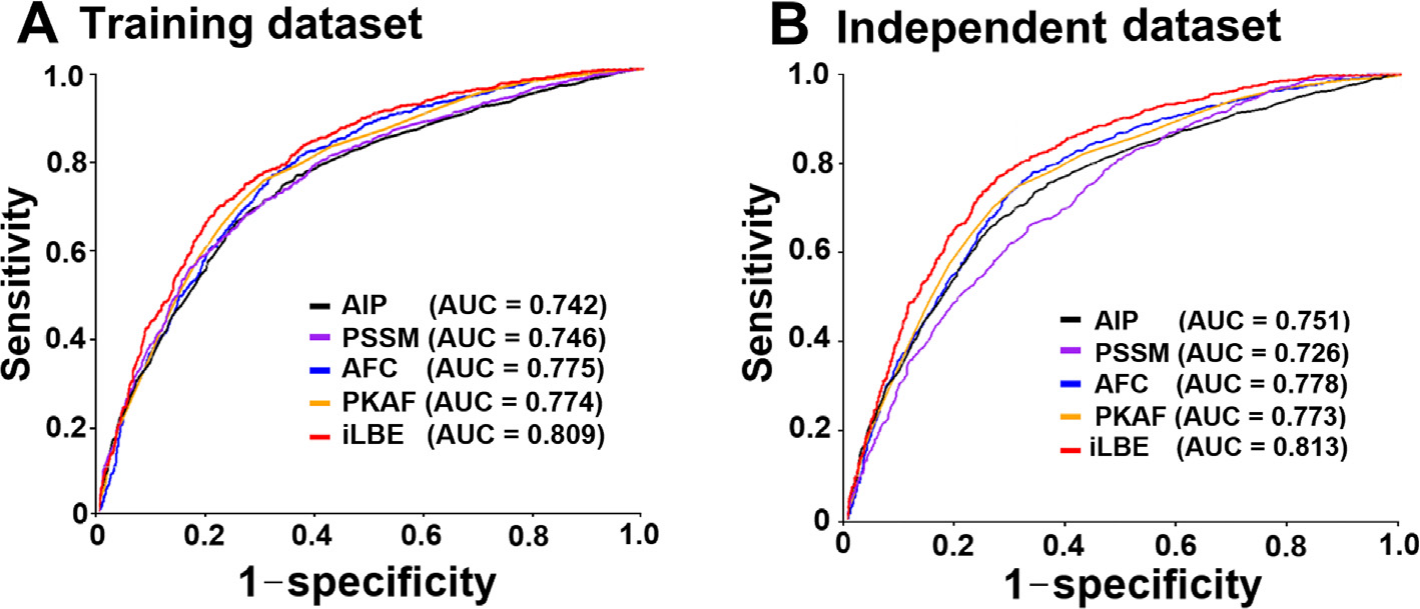
**ROC curves of various prediction models** **A.** and **B.** ROC curves for the four encoding schemes PSSM, AIP, PKAF, and AFC, as well as their LR-combined iLBE model, are presented for the training dataset (A) and independent dataset (B). The LR coefficients for PSSM, AIP, PKAF, and AFC are 0.435, 0.102, 1.337, and 0.465, respectively. ROC, receiver operating characteristic.

The performances of each single feature vector-trained model and the combined model were evaluated in the training and independent datasets, as shown in Figure 3. AUCs obtained using iLBE were higher than those obtained using any single feature model for both training and independent datasets, demonstrating the robustness of the iLBE model. Moreover, we also measured the predictive performance based on either sequence or evolutionary features alone for the training and independent datasets (Table S1). The AUC values of the sequence feature-based methods were at most 0.791 and 0.798 for the training and independent datasets, respectively (Table S1). Similarly, the AUC values of the evolutionary feature-based methods were at most 0.789 and 0.786 for the training and independent datasets, respectively. Neither the sequence nor evolutionary feature-based methods outperformed iLBE, indicating that the combination of the sequence and evolutionary features in iLBE is effective for enhanced prediction accuracy.

In addition, we used BLAST to determine the sequence profile information of BCEs and non-BCEs in the training dataset [40]. In total 1038 BCE and 597 non-BCE samples were selected out of 4440 BCE and 5485 non-BCE samples via the BLASTP with an E-value of 0.01. Then the BLAST performance was evaluated through a 10-fold CV test. The Sn, Ac, MCC, and AUC were 0.214, 0.544, 0.042, and 0.569, respectively, which are lower than those of iLBE. Therefore, BLAST was not considered for the final prediction.

**Figure 4:**
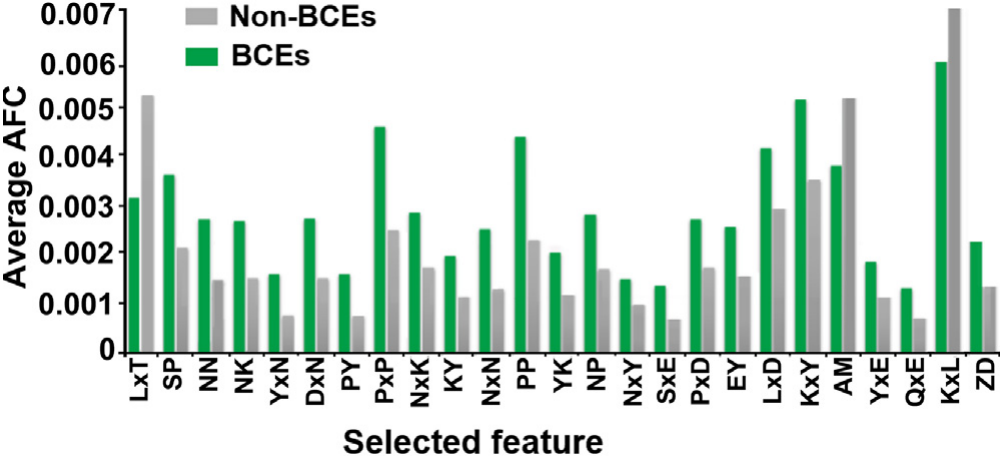
**Distribution of the top 25 significant features derived from the AFC scheme** The Y-axis represents the average AFC values for BCEs and non-BCEs. The X-axis represents the selected features of amino acid pair. The letter x represent any amino acid.

We found that the AFC scheme presented the highest AUC, Sn, Ac, and MCC for all four single encoding methods (Table 2). To investigate significant residues estimated by the AFC method, the top 25 amino acid pairs were examined through the WR feature selection. The top 25 significant residue pairs and corresponding *P* values are listed in Table S2. As shown in Figure 4, the average AFC value was measured for BCEs and non-BCEs. The selected feature of LxT (where ‘x’ signifies any amino acid) was the most significant residue pair and depleted around non-BCE (*P* = 3.112E–12, paired two-sample *t*-test, Table S2). Likewise, the feature SP that characterizes a 0-spaced (*i.e.*, there is no space in this case) pair of residues SP is important and enriched in BCEs (Figure 4; *P* = 2.88E–09, paired two-sample *t*-test, Table S2). The above similar concept was applied to other selected pairs of residues (Figure 4). Importantly, the top 25 features contained P, N, and Y residues, which showed strong preference in positional residue analysis (Figure 2). These residues would play an important role in the recognition of BCEs. Moreover, as shown in Table S2, the average AFC values of top 25 features were significantly different between BCEs and non-BCEs (*P* < 0.05; paired two-sample *t*-test).

### Optimal length of epitopes

To optimize the length of short epitopes, we investigated the different lengths (5, 10, 15, 20, or 25 amino acids) of BCEs using the four encoding schemes of AIP, PSSM, AFC, and PKAF and their combined scheme (iLBE) (Table S3). The RF algorithm without any feature selection approach was used to evaluate prediction performance on the training data via a 10-fold CV test. The prediction performance increased with an increase in sequence length, and was saturated for lengths of 20 and 25 (Table S3). Therefore, a sequence length of 24 was determined for iLBE.

### Comparison of RF with **other widely-used** ML algorithms

The RF algorithm was characterized in comparison with the widely-used ML algorithms of NB, SVM, and ANN on the same training dataset. AUC values of predictions using the four algorithms without any feature selection were evaluated by a 10-fold CV test. As shown in Table S4, the RF algorithm provided a higher AUC than any other algorithms. Accordingly, we implement the RF algorithm in iLBE.

### Comparison of iLBE with existing methodologies

We evaluated the prediction performance of the proposed iLBE with existing approaches on the same dataset. First, we employed the training dataset to compare the performance of iLBE with those of the LBtope and iBCE-EL models, which are the state-of-the-art predictors and publicly accessible. As shown in Table 3, an increase in Sp decreased Sn for iLBE. iLBE with the moderate threshold showed higher Sp, Sn, MCC, Ac, and AUC than LBtope and iBCE-EL, demonstrating that iLBE outperforms the existing pioneering predictors. Furthermore, we compared the performance of iLBE with those of LBtope and iBCE-EL in the independent dataset (see Method). As shown in Table 4, an increase in Sp also decreased Sn for iLBE in the independent dataset. iLBE with the moderate threshold outperformed the two existing methods in terms of Sp, MCC, Ac, and AUC, while it presented almost the same Sn as LBtope. The superiority of iLBE to the existing methods was confirmed to be significant (*P* < 0.05, paired two sample *t*-test).

**Table 3:** **Performance comparison between iLBE and existing predictors in the training dataset**

**Predictor**	**Threshold**	**Sp**	**Sn**	**Ac**	**MCC**	**AUC**
LBtope	–	0.672	0.660	0.667	0.330	0.730
iBCE-EL	–	0.739	0.716	0.729	0.454	0.782
iLBE	High	0.866	0.568	0.733	0.452	0.809
	Moderate	0.747	0.759	0.752	0.496	0.809
	Low	0.636	0.838	0.726	0.475	0.809

*Note*: A 10-fold CV test was applied to the training dataset. The performances of LBtope and iBCE-EL were collected according to their published studies [28], [34]. In the proposed iLBE, the high-, moderate-, and low-level thresholds were determined based on the RF scores of 0.485, 0.410, and 0.360, respectively, which corresponded to the Sp levels of 0.866, 0.747, and 0.636, respectively, in the training dataset.

**Table 4:** **Performance comparison between iLBE and existing predictors in the independent dataset**

**Predictor**	**Threshold**	**Sp**	**Sn**	**Ac**	**MCC**	**AUC**	***P*****value**
LBtope	–	0.567	0.759	0.615	0.328	0.730	< 0.01
iBCE-EL	–	0.724	0.742	0.732	0.463	0.786	< 0.05
iLBE	High	0.861	0.554	0.726	0.440	0.813	
	Moderate	0.745	0.752	0.748	0.494	0.813	
	Low	0.635	0.830	0.721	0.467	0.813	

*Note*: The high-, moderate-, and low-level thresholds for iLBE were considered based on the training dataset performance. Significant difference between iLBE and the respective existing method was analyzed using a paired two-sample *t*-test based on the AUC values (*P* < 0.05).

### Effect of combination methods

To investigate the effects of combination methods on the prediction performance, we built a competitive model of iLBE, which arranges the four encoding vectors of AFC, AIP, PSSM, and PKAF in a row, instead of the use of LR. It is named as the sequential combination model. The resultant total dimension was 2192. The top 380 feature vectors were collected and rearranged in the ascending order of WR values. The WR-optimized feature vectors were used to train the RF classifier via a 10-fold CV test. The sequential combination model with and without feature collection approaches yielded AUC values of 0.778 and 0.767 on the training dataset, respectively (Figure S1A), and presented 0.798 and 0.781 on the independent dataset, respectively **(**Figure S1B). The LR-based combination of iLBE outperformed the sequential combination model (Figure 3) and was found to be the best in this study.

## Conclusion

We have developed a novel computational predictor, iLBE, which accurately predicts BCEs for both the training and independent datasets. iLBE outperformed existing state-of-the-art predictors LBtope and iBCE-EL. The iLBE model combined the sequence-based features and evolutionary information, while the LBtope and iBCE-EL predictors only used sequence-based encoding methods. iLBE employed the LR-based combined model of the RF-based classifiers, while LBtope and iBCE-EL used SVM and an ensemble ML model, respectively. Importantly, iLBE allows the use of various threshold values at high, moderate, and low levels to demonstrate whether a BCE is highly positive or negative, which is not available in the existing prediction tools. As a complementary to the experimental strategies, iLBE provides insight into the functional and significant characteristics of BCEs. A user-friendly web-application was also developed for easy use by the immunological research community.

## Availability

A web application with curated datasets for iLBE is freely accessible at http://kurata14.bio.kyutech.ac.jp/iLBE/.

## CRediT author statement

**Md. Mehedi Hasan:** Conceptualization, Data curation, Methodology, Formal analysis, Software, Writing - original draft. **Mst. Shamima Khatun:** Data curation, Formal analysis, Methodology, Software. **Hiroyuki Kurata:** Conceptualization, Supervision, Writing - original draft. All authors read and approved the final manuscript.

## Competing interests

The authors have declared no competing interests.

## Supplementary data

Supplementary Figure S1 **ROC curves of the sequential combination model that integrates the feature vectors with and without feature selection approach**. **A.** Training dataset. **B.** Independent dataset.

Supplementary Table S1 **AUC values of prediction based on sequence or evolutionary features**

Supplementary Table S2 **Top 25 AFC features ranked by a WR-based selection method**

Supplementary Table S3 **AUC values for different lengths of epitopes**

Supplementary Table S4 **AUC values for different ML algorithms**
