# Supplementary figures and images for: iLBE for Computational Identification of Linear B-cell Epitopes by Integrating Sequence and Evolutionary Features
Source: Genomics Proteomics Bioinformatics. 2020 Oct 22;18(5):593–600. doi: 10.1016/j.gpb.2019.04.004 (PMC8377379; doi:10.1016/j.gpb.2019.04.004)

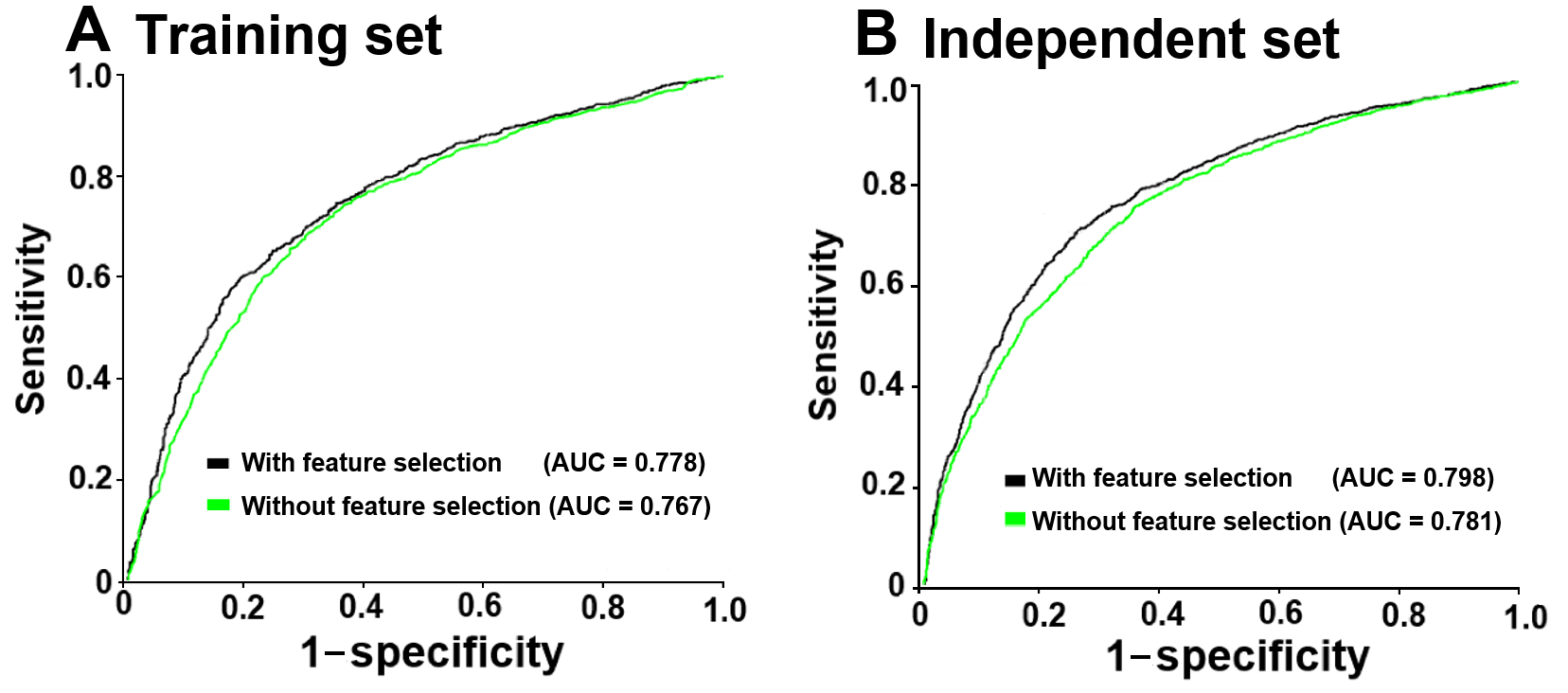

Supplement: Supplementary Figure S1 — ROC curves of the sequential combination model that integrates the feature vectors with and without feature selection approach. A. Training dataset. B. Independent dataset. [file mmc1.zip › Figure S1 Au120919.png]
