# Supplementary material for: iLBE for Computational Identification of Linear B-cell Epitopes by Integrating Sequence and Evolutionary Features
Source: Genomics Proteomics Bioinformatics. 2020 Oct 22;18(5):593–600. doi: 10.1016/j.gpb.2019.04.004 (PMC8377379; doi:10.1016/j.gpb.2019.04.004)
Supplement: Supplementary Table S1 — AUC values of prediction based on sequence or evolutionary features [file mmc2.docx]

**Table S2 Top 25 AFC features ranked by a WR-based selection method**

| **No. of feature** | **WR feature** | ***P* value** |
| --- | --- | --- |
| 1  2  3  4  5  6  7  8  9  10  11  12  13  14  15  16  17  18  19  20  21  22  23  24  25 | L×T  SP  NN  NK  Y×N  D×N  PY  P×P  N×K  KY  N×N  PP  YK  NP  N×Y  S×E  P×D  EY  L×D  K×Y  AM  Y×E  Q×E  K×L  ND | 3.112E-12  2.88E-09  4.76E-08  1.29E-08  2.91E-08  9.39E-09  9.18E-08  1.28E-08  2.82E-07  1.03E-06  6.77E-08  1.76E-07  2.51E-06  6.09E-06  7.01E-06  4.04E-06  4.17E-06  1.28E-06  3.34E-06  3.068E-06  7.75E-06  9.32E-06  1.21E-05  1.78E-05  3.39E-04 |

*Note*: *P* values were calculated using a paired *t*-test for the top 25 significant BCEs and non-BCEs.
