# Supplementary material for: iLBE for Computational Identification of Linear B-cell Epitopes by Integrating Sequence and Evolutionary Features
Source: Genomics Proteomics Bioinformatics. 2020 Oct 22;18(5):593–600. doi: 10.1016/j.gpb.2019.04.004 (PMC8377379; doi:10.1016/j.gpb.2019.04.004)
Supplement: Supplementary Table S2 — Top 25 AFC features ranked by a WR-based selection method [file mmc3.docx]

**Table S2 Top 25 AFC features ranked by a WR-based selection method**

| **Feature No.** | **WR feature** | ***P* value** |
| --- | --- | --- |
| 1 | LxT | 3.112E–12 |
| 2 | SP | 2.88E–09 |
| 3 | NN | 4.76E–08 |
| 4 | NK | 1.29E–08 |
| 5 | YxN | 2.91E–08 |
| 6 | DxN | 9.39E–09 |
| 7 | PY | 9.18E–08 |
| 8 | PxP | 1.28E–08 |
| 9 | NxK | 2.82E–07 |
| 10 | KY | 1.03E–06 |
| 11 | NxN | 6.77E–08 |
| 12 | PP | 1.76E–07 |
| 13 | YK | 2.51E–06 |
| 14 | NP | 6.09E–06 |
| 15 | NxY | 7.01E–06 |
| 16 | SxE | 4.04E–06 |
| 17 | PxD | 4.17E–06 |
| 18 | EY | 1.28E–06 |
| 19 | LxD | 3.34E–06 |
| 20 | KxY | 3.068E–06 |
| 21 | AM | 7.75E–06 |
| 22 | YxE | 9.32E–06 |
| 23 | QxE | 1.21E–05 |
| 24 | KxL | 1.78E–05 |
| 25 | ND | 3.39E–04 |

*Note*: The *P* values were calculated using a paired *t*-test for the top 25 significant BCEs and non-BCEs.
