# Supplementary material for: iLBE for Computational Identification of Linear B-cell Epitopes by Integrating Sequence and Evolutionary Features
Source: Genomics Proteomics Bioinformatics. 2020 Oct 22;18(5):593–600. doi: 10.1016/j.gpb.2019.04.004 (PMC8377379; doi:10.1016/j.gpb.2019.04.004)
Supplement: Supplementary Table S3 — AUC values for different lengths of epitopes [file mmc4.docx]

**Table S3** **AUC values for different lengths of epitopes**

| **Length** | **AIP** | **PSSM** | **AFC** | **PKAF** | **iLBE** |
| --- | --- | --- | --- | --- | --- |
| 5 aa | 0.526 | 0.538 | 0.546 | 0.555 | 0.563 |
| 10 aa | 0.557 | 0.559 | 0.579 | 0.576 | 0.598 |
| 15 aa | 0.589 | 0.588 | 0.663 | 0.687 | 0.718 |
| 20 aa | 0.703 | 0.737 | 0.765 | 0.761 | 0.781 |
| 25 aa | 0.716 | 0.729 | 0.758 | 0.763 | 0.786 |

*Note*: A 10-fold CV test was applied to the training dataset. aa, amino acid.
