# Supplementary material for: iLBE for Computational Identification of Linear B-cell Epitopes by Integrating Sequence and Evolutionary Features
Source: Genomics Proteomics Bioinformatics. 2020 Oct 22;18(5):593–600. doi: 10.1016/j.gpb.2019.04.004 (PMC8377379; doi:10.1016/j.gpb.2019.04.004)
Supplement: Supplementary Table S4 — AUC values for different ML algorithms [file mmc5.docx]

**Table S4** **AUC values for different ML algorithms**

| Algorithm | PSSM | AIP | PKAF | AFC | iLBE |
| --- | --- | --- | --- | --- | --- |
| NB | 0.682 | 0.717 | 0.736 | 0.747 | 0.756 |
| ANN | 0.699 | 0.711 | 0.732 | 0.739 | 0.743 |
| SVM | 0.733 | 0.721 | 0.753 | 0.766 | 0.774 |
| RF | 0.738 | 0.739 | 0.768 | 0.767 | 0.788 |

*Note*: A 10-fold CV test was applied to the training dataset. NB, naive Bayes; ANN, artificial neural network; SVM, support vector machine; RF, random forest.
